# Supplementary material for: Universal microbial reworking of dissolved organic matter along environmental gradients
Source: Nat Commun. 2024 Jan 2;15:187. doi: 10.1038/s41467-023-44431-4 (PMC10762207; doi:10.1038/s41467-023-44431-4)
Supplement: Supplementary file 1 — Supplementary Information [file 41467_2023_44431_MOESM1_ESM.pdf]

Supplementary Information: Universal microbial reworking of dissolved organic matter along environmental gradients

Erika C Freeman[1,*], Erik JS Emilson[2,3], Thorsten Dittmar[4,5], Lucas PP Braga[1], Caroline E Emilson[2], Tobias Goldhammer[6], Christine Martineau[7], Gabriel Singer[8], Andrew J Tanentzap[1,3]

[1]Ecosystems and Global Change Group, Department of Plant Sciences, University of Cambridge, Cambridge CB2 3EA, United Kingdom

[2]Natural Resources Canada, Canadian Forest Service, Great Lakes Forestry Centre, 1219 Queen St. E., Sault Ste. Marie, ON, P6A 2E5, Canada

[3]Ecosystems and Global Change Group, School of the Environment, Trent University, Peterborough, ON K9L 0G2, Canada

[4]Institute for Chemistry and Biology of the Marine Environment, University of Oldenburg, 26129 Oldenburg, Germany

[5]Helmholtz Institute for Functional Marine Biodiversity, University of Oldenburg, 26129 Oldenburg, Germany

[6]Department of Ecohydrology and Biogeochemistry, Leibniz-Institute of Freshwater Ecology and Inland Fisheries, Mueggelseedamm 301, Berlin, Germany

[7]Natural Resources Canada, Laurentian Forestry Centre, 1055 Du P.E.P.S. Street, P.O. Box 10380, Québec, G1V 4C7, Canada

[8]Department of Ecology, University of Innsbruck, Technikerstrasse 25, 6020 Innsbruck, Austria

This .pdf file includes:
Supplementary Figures 1 – 5
Supplementary Tables 1 – 13
Supplementary Results
Supplementary Methods
Supplementary References

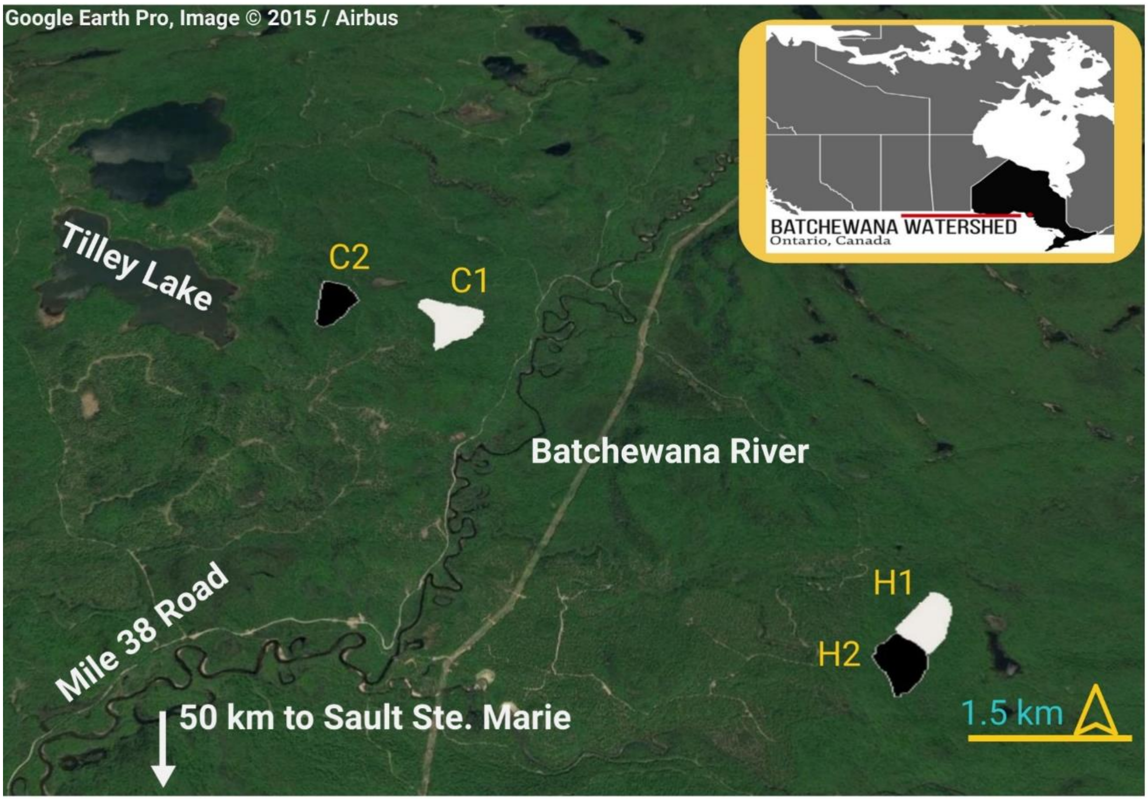

Figure S1: Study area within Batchewana watershed, central Ontario, Canada with four replicate catchments named C1, C2, H1, H2. These catchments were recruited as part of a forest harvest experiment and the names reflect this design: C = control and H = harvest. All samples were taken prior to these catchments being harvested and therefore can be treated as replicates. All geographic coordinates for sampling points can be accessed and downloaded from: https://www.gaiagps.com/public/VKMlEPlvf1t6ownd6Vulfm8T. Map data: Google, ©2015 / Airbus satellite data.

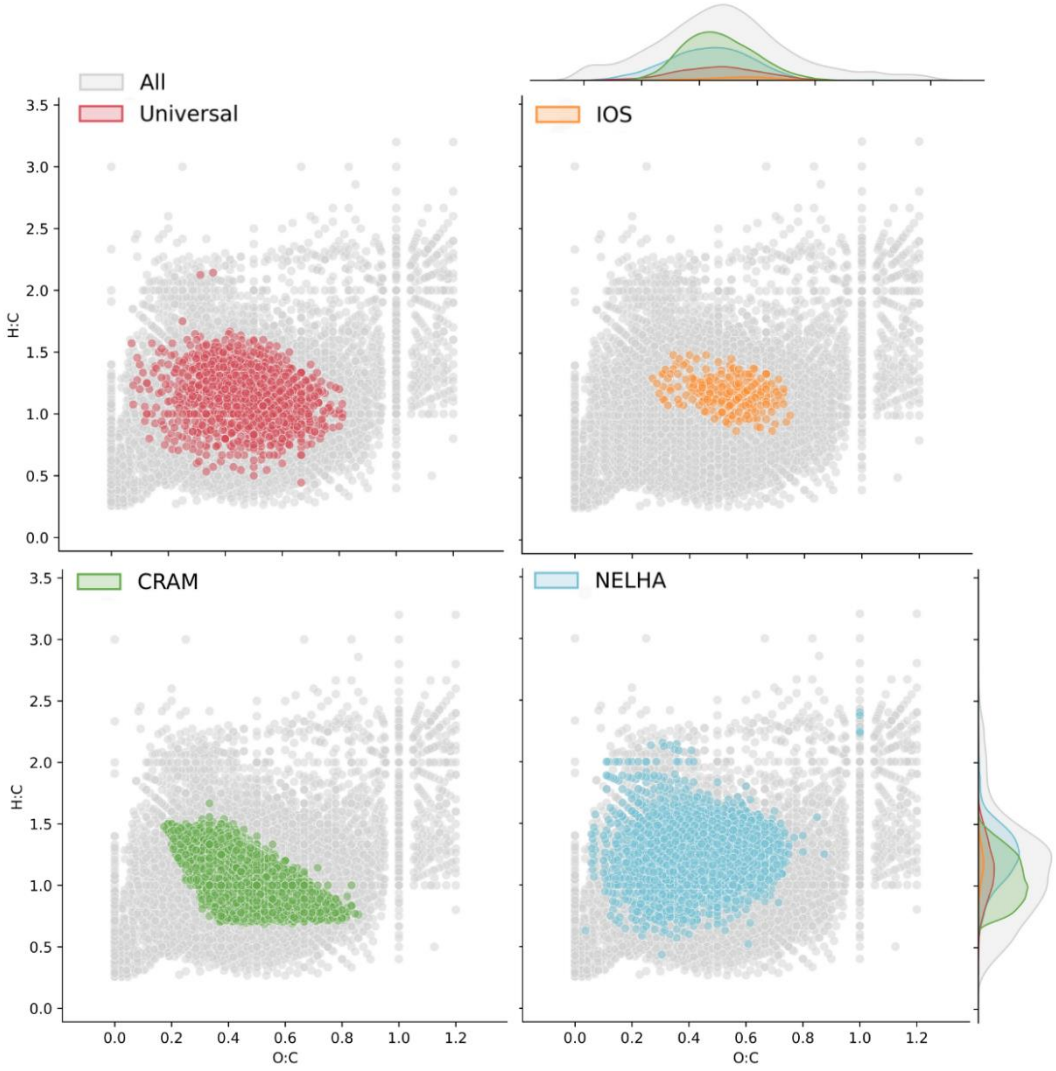

Figure S2: Molecular properties are shared by different definitions of degradation end-products that should be universally distributed. Each point is the elemental ratio (hydrogen to carbon and oxygen to carbon) of a molecular formula detected in our study ("All" category, n=9327). We further coloured these points according to whether they were present in every soil and stream sample in this study ("Universal"; n=1216), carboxyl-rich alicyclic molecules (CRAM, n = 3735), found in a subset of molecular formulae representing aged, degradation end-products identified in marine environments[1,2] (IOS, n=233), or found in a deep-sea reference sample from the Natural Energy Laboratory of Hawaii Authority (NEHLA) facility and expected to be dominated by degradation end-products (n=3019). CRAM were defined as molecular formulae that contained double bond equivalent (DBE) to C ratios of 0.30 to 0.68, DBE to H ratios of 0.20 to 0.95, and DBE to O ratios of 0.77 to 1.75[3]. Marginal axes show density curves for each compound category.

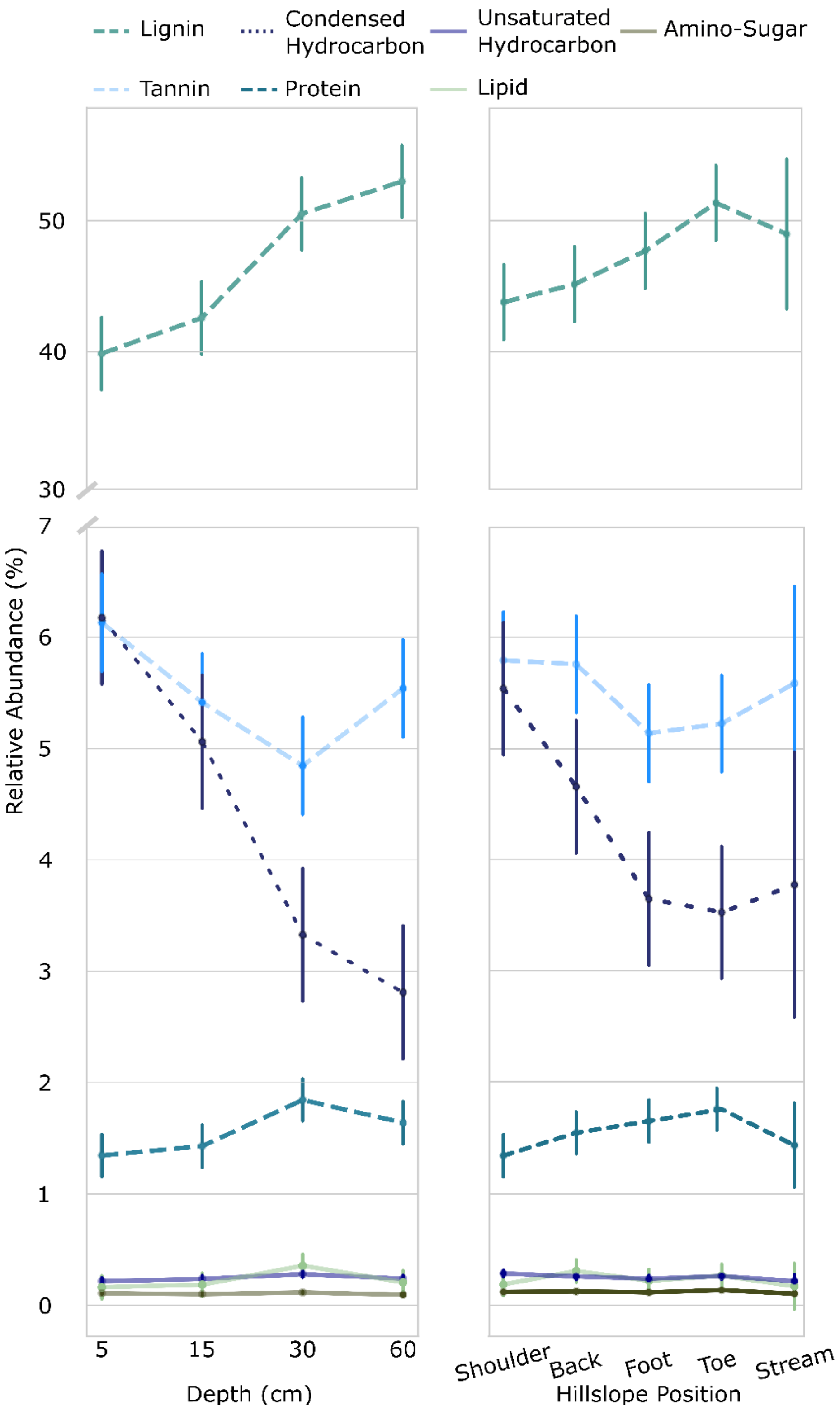

Figure S3: Compound classes within the universal pool shift in relative abundance across soil depth and hillslope position. Mean estimated molecular mass (± 95% CI) of dissolved organic matter for each compound class when only universal compounds were considered. Molecular formulae were grouped into compound classes based on their atomic ratios. Darkened lines are those with statistically significant differences between 5 and 60 cm depths or shoulder and stream positions based on linear models (Table S4). The Tukey method was used to adjust p values for multiple comparisons.

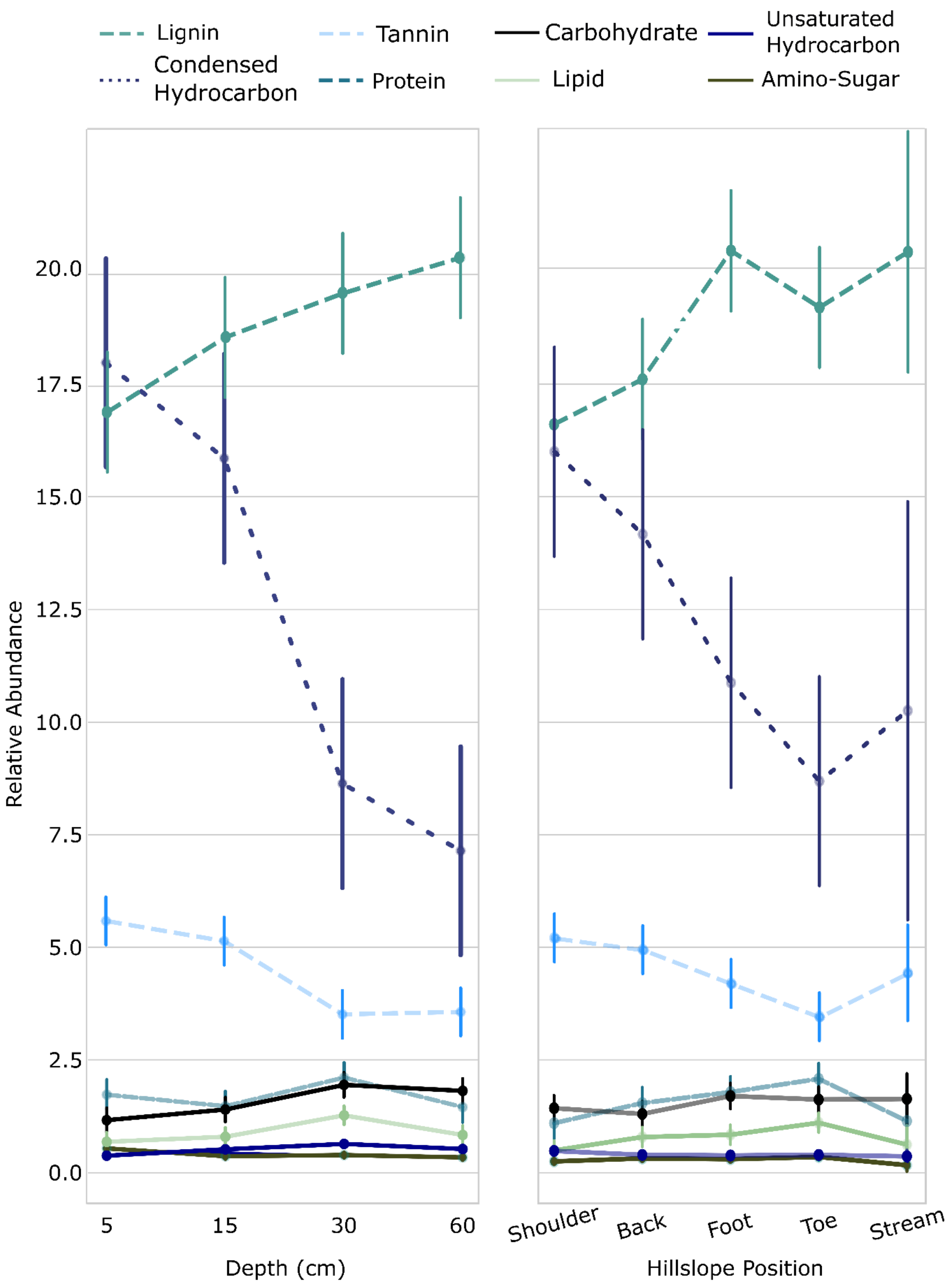

Figure S4: Compound classes within the non-universal pool shift in relative abundance across soil depth and hillslope position. Mean estimated molecular mass (± 95% CI) of dissolved organic matter for each compound class when only universal compounds were considered. Molecular formulae were grouped into compound classes based on their atomic ratios. Darkened lines are those with statistically significant differences between 5 and 60 cm depths or shoulder and stream positions based on linear models (Table S5). The Tukey method was used to adjust p values for multiple comparisons.

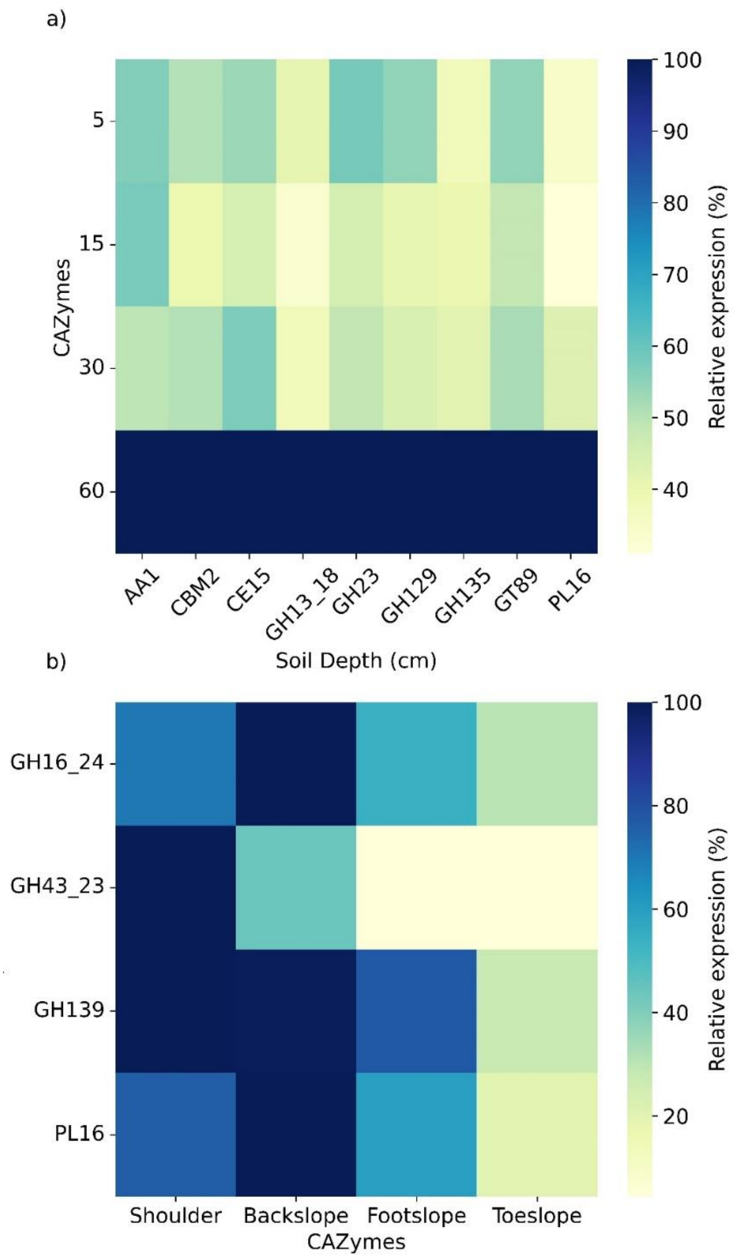

Figure S5: Expression of genes annotated as carbohydrate-active enzymes (CAZymes) across (a) depth and (b) hillslope positions. We only plotted those genes that varied from the start to end of the spatial gradients based on a mixed-effect model (Table S9). For each gene, we scaled expression relative to the maximum level observed for that gene at each depth or hillslope position.

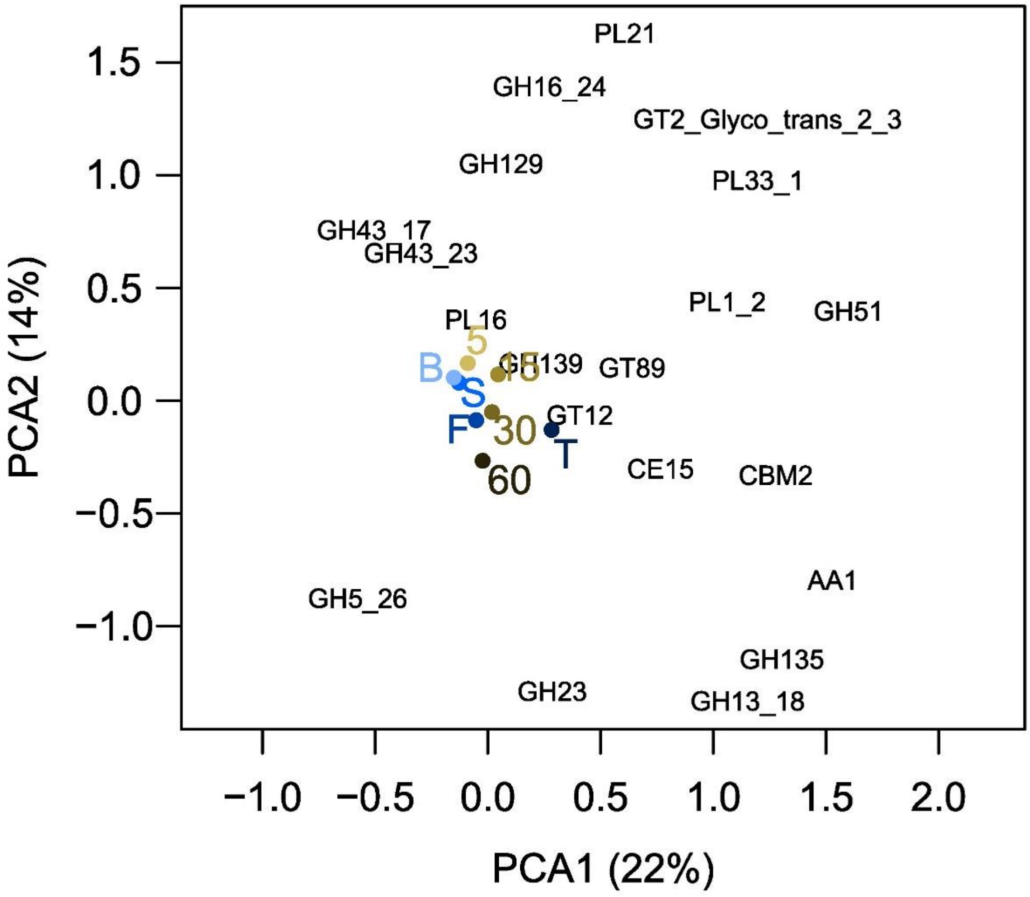

Figure S6: Expression of carbohydrate-active enzymes (CAZymes) shifted with depth and hillslope position. We used principal components analysis (PCA) to reduce the variation in expression of 20 CAZymes onto two dimensions, with variation explained by each PCA axis shown in parentheses. The 20 CAZymes were those that explained variation in non-universal compound classes that increased in molecular mass along across the depth and hillslope gradients, i.e., bolded classes in Fig 4b. Positions of individual CAZymes plotted in black text with abbreviations defined in Table S9. Points correspond with centroids for each depth from 5 to 60cm and hillslope position: S=shoulder, B=backslope, F=footslope, T=toeslope.

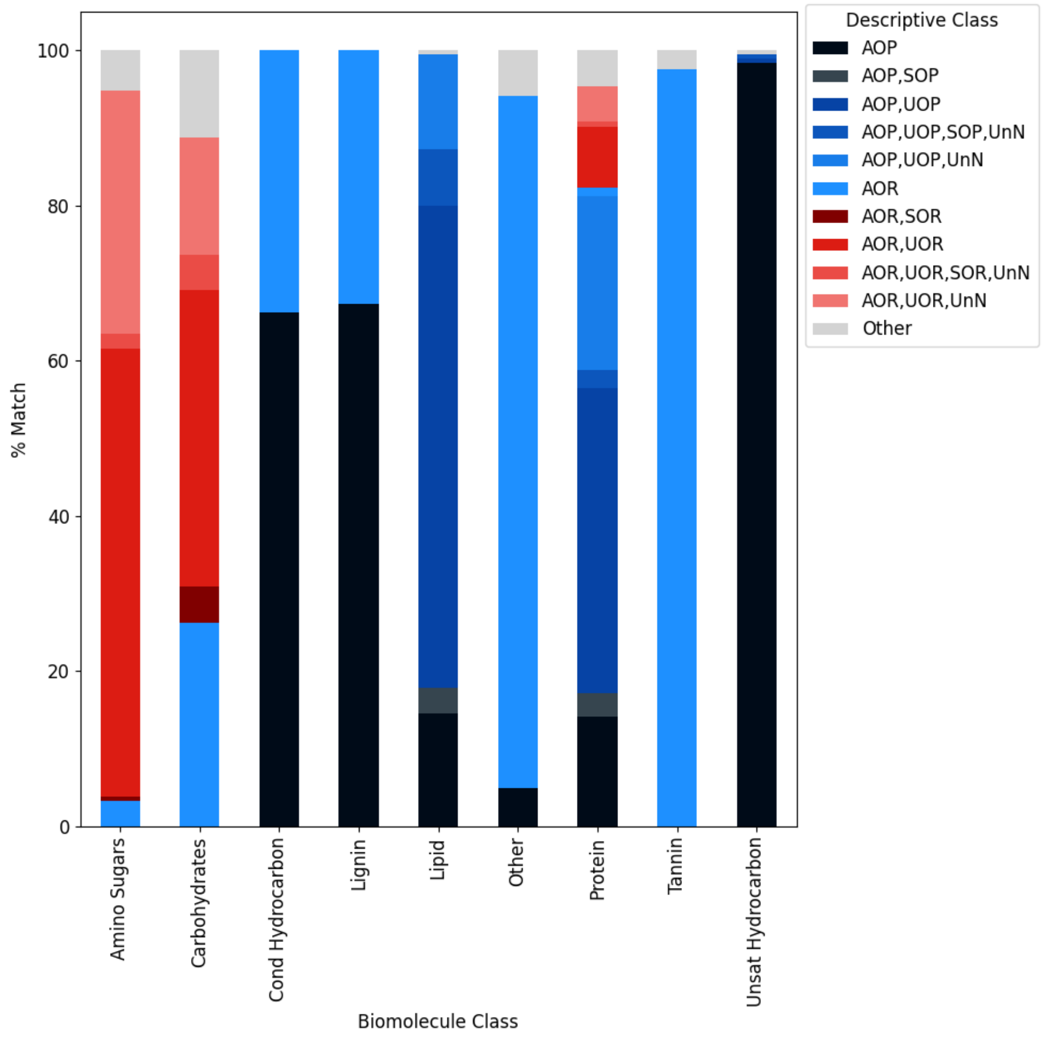

Figure S7: Comparison of compound classifications. We reclassified each molecular formula assigned to a compound class in the Main Text after ref. [4] using the descriptive classes of ref. [5]. Ref. [5] incorporates additional information on the modified aromaticity index and double bond equivalents of each formula in addition to the H:C and O:C ratios used by ref. [4]. The resulting descriptive classes can overlap and include: AOP = aromatic, oxygen-poor, SOP = saturated oxygen-poor, UOP = unsaturated oxygen-poor, UnN= unsaturated with N, SOR= saturated oxygen-poor or Other = unclassified.

Table S1: Size-exclusion chromatography indicates high-molecular-weight substances (HMWS) outside the measurement size of ultra-high-resolution mass spectrometry were not abundant in our samples. CDOC = Dissolved organic carbon determined after chromatography. All measurements in mg C L^{-1}. "-" in HMWS concentrations indicates below limit of quantification (<0.5 mg C L^{-1}). % C pool refers to the % HMWS C in the CDOC pool.

Site	Hillslope Position	Depth (cm)	CDOC	HMWS	% C pool
C1	Shoulder	5	27.10	-	
C1	Shoulder	15	22.70	0.67	2.95
C1	Shoulder	30	17.70	-	
C1	Shoulder	60	26.57	1.49	5.61
C1	Backslope	5	40.20	0.52	1.29
C1	Backslope	15	35.90	-	
C1	Backslope	30	7.40	-	
C1	Backslope	60	10.60	-	
C1	Footslope	5	25.00	-	
C1	Footslope	15	9.90	-	
C1	Footslope	30	5.10	1.56	30.58
C1	Footslope	60	2.70	-	
C1	Toeslope	5	14.90	-	
C1	Toeslope	15	8.60	-	
C1	Toeslope	30	6.40	-	
C2	Shoulder	5	42.80	-	
C2	Shoulder	15	52.60	-	
C2	Shoulder	30	19.68	-	
C2	Shoulder	60	8.79	-	
C2	Backslope	5	31.80	-	
C2	Backslope	15	23.30	-	
C2	Backslope	30	17.60	1.2	6.82
C2	Backslope	60	6.70	-	
C2	Footslope	5	20.07	-	
C2	Footslope	15	11.30	-	
C2	Footslope	30	5.00	-	
C2	Footslope	60	3.60	-	
C2	Toeslope	5	7.80	-	
C2	Toeslope	15	3.80	-	
C2	Toeslope	30	5.90	-	
C2	Toeslope	60	1.50	-	
H1	Shoulder	5	15.10	-	
H1	Shoulder	15	20.87	-	
H1	Shoulder	30	9.35	-	
H1	Shoulder	60	8.30	-	
H1	Backslope	5	14.87	-	
H1	Backslope	15	14.40	-	

H1	Backslope	30	5.80	-	
H1	Backslope	60	4.70	-	
H1	Footslope	5	11.70	0.53	4.53
H1	Footslope	15	9.40	-	
H1	Footslope	30	4.30	-	
H1	Footslope	60	2.18	-	
H1	Toeslope	5	2.40	-	
H1	Toeslope	15	1.63	-	
H1	Toeslope	30	1.90	-	
H1	Toeslope	60	1.45	-	
H2	Shoulder	5	23.30	-	
H2	Shoulder	15	27.50	-	
H2	Shoulder	30	2.00	-	
H2	Shoulder	60	5.32	-	
H2	Backslope	5	14.20	-	
H2	Backslope	15	8.40	0.44	5.23
H2	Backslope	30	7.20	-	
H2	Backslope	60	8.30	0.53	6.39
H2	Footslope	5	3.54	-	
H2	Footslope	15	3.60	-	
H2	Footslope	30	3.45	-	
H2	Footslope	60	1.49	-	
H2	Toeslope	5	6.49	-	
H2	Toeslope	15	3.65	-	
H2	Toeslope	30	1.92	-	
H2	Toeslope	60	1.37	-	
HMWS detected (% samples)				13 (8 of 63)	

Table S2: Linear models predicting the number and relative abundance of universal compounds. We modelled the proportion of molecular formulae that were counted in every sample (n=1216), along with their relative abundance (i.e. summed normalised signal intensity). We compared these with the proportion of molecular formulae and relative abundance that overlapped with the "Island of Stability" (IOS, n=233) – a literature-based definition of universal-like molecular formulae[1,2,6] (Fig. S2). We also modelled the relative abundance of compounds in our samples that could be defined as carboxyl-rich alicyclic molecules (CRAM), which contain double bond equivalent (DBE) to C ratios of 0.30 to 0.68, DBE to H ratios of 0.20 to 0.95, and DBE to O ratios of 0.77 to 1.75[3] (n=3735), and compounds found in a deep-sea reference (n=3019) collected near the Natural Energy Laboratory of Hawaii Authority (NEHLA) facility, which should be dominated by widely distributed, degradation end-products. Values in cells are mean estimated effects ± standard error relative to the intercept, with the intercept expressed relative to zero. Proportions were modelled using a binomial error structure, so effects are expressed on a logit-scale and correspond with odds ratios. Bolded values were statistically significant at ***p < 0.001, **p < 0.01, *p < 0.05. Adjusted R^2 for linear models with binomial error structures were estimated using the MuMIn package in R[7]. For all, degrees of freedom = 57.

Coefficient	**Proportion of All Compounds**	**Abundance of All Compounds**	**Proportion IOS**	**Abundance of IOS**	**Abundance of CRAM**	**Abundance of NELHA**
Intercept: Site C1, 5 cm depth, shoulder position	**0.26 *** (0.03)**	**0.59 ** (0.06)**	**0.04 *** (0.00)**	**0.20 *** (0.02)**	**0.58 *** (0.04)**	**0.62 ** (0.11)**
Site C2	**1.19 * (0.10)**	0.53 (0.04)	**1.14 * (0.07)**	0.51 (0.03)	0.51 (0.02)	0.53 (0.06)
Site H1	1.12 (0.09)	0.51 (0.03)	1.09 (0.07)	0.52 (0.03)	**0.52 * (0.02)**	0.54 (0.06)
Site H2	1.05 (0.09)	0.50 (0.03)	1.05 (0.07)	0.52 (0.03)	**0.52 * (0.02)**	0.54 (0.06)
15 cm	1.00 (0.08)	0.51 (0.04)	1.00 (0.06)	0.52 (0.03)	0.52 (0.02)	0.54 (0.06)
30 cm	1.14 (0.10)	**0.57 *** (0.04)**	1.11 (0.07)	**0.59 *** (0.04)**	**0.56 *** (0.03)**	**0.65 *** (0.07)**
60 cm	**1.19 * (0.10)**	**0.60 *** (0.04)**	**1.14 * (0.07)**	**0.63 *** (0.04)**	**0.59 *** (0.03)**	**0.67 *** (0.07)**
Backslope	1.01 (0.09)	0.51 (0.04)	1.01 (0.07)	0.52 (0.03)	0.52 (0.02)	0.53 (0.06)
Footslope	0.99 (0.08)	0.51 (0.04)	0.99 (0.06)	**0.55 ** (0.04)**	**0.55 *** (0.03)**	**0.59 ** (0.06)**
Toeslope	1.16 (0.10)	**0.55 ** (0.04)**	1.12 (0.07)	**0.57 *** (0.04)**	**0.57 *** (0.03)**	**0.63 *** (0.07)**
Stream	0.90 (0.13)	**0.43 * (0.05)**	0.92 (0.10)	**0.38 *** (0.04)**	**0.40 *** (0.03)**	**0.33 *** (0.06)**
R^2	0.20	0.42	0.21	0.59	0.64	0.54

Table S3: Linear models predicting the relative abundance of classes within the universal compound pool. Carbohydrates were not found in universal compounds and are omitted from this table. Values in cells are mean estimated effects ± standard error relative to the intercept, with the intercept expressed relative to zero. Bolded values were statistically significant at ***p < 0.001, **p < 0.01, *p < 0.05. For all, degrees of freedom = 57.

	Lignins	Tannins	Condensed Hydrocarbons	Proteins	Amino Sugars	Lipids	Unsaturated Hydrocarbons
Coefficient	*Estimates*						
Intercept: Site C1, 5 cm depth, shoulder position	**47.12 *** (2.92)**	**5.62 *** (0.47)**	**4.35 *** (0.65)**	**1.37 *** (0.21)**	**0.09 *** (0.01)**	0.12 (0.11)	**0.20 *** (0.03)**
Site C2	2.93 (1.84)	-0.16 (0.30)	-0.22 (0.41)	0.17 (0.13)	0.00 (0.01)	0.03 (0.07)	0.04 (0.02)
Site H1	1.43 (1.84)	-0.03 (0.30)	**-0.96 * (0.41)**	0.08 (0.13)	-0.01 (0.01)	0.03 (0.07)	0.00 (0.02)
Site H2	0.96 (1.84)	0.07 (0.30)	**-1.12 ** (0.41)**	-0.02 (0.13)	-0.01 (0.01)	0.09 (0.07)	-0.01 (0.02)
15 cm	2.66 (1.90)	**-0.72 * (0.31)**	**-1.11 * (0.42)**	0.08 (0.13)	-0.01 (0.01)	0.02 (0.07)	0.02 (0.02)
30 cm	**10.41 *** (1.90)**	**-1.28 *** (0.31)**	**-2.85 *** (0.42)**	**0.50 *** (0.13)**	0.01 (0.01)	**0.19 * (0.07)**	**0.06 ** (0.02)**
60 cm	**12.83 *** (1.90)**	-0.59 (0.31)	**-3.36 *** (0.42)**	**0.29 * (0.13)**	-0.01 (0.01)	0.04 (0.07)	0.02 (0.02)
Backslope	1.29 (1.90)	-0.04 (0.31)	-0.88 * (0.42)	0.21 (0.13)	0.00 (0.01)	0.12 (0.07)	-0.03 (0.02)
Footslope	3.68 (1.90)	**-0.66 * (0.31)**	**-1.90 *** (0.42)**	**0.31 * (0.13)**	-0.00 (0.01)	0.03 (0.07)	**-0.05 * (0.02)**
Toeslope	**7.14 *** (1.90)**	-0.57 (0.31)	**-2.02 *** (0.42)**	**0.42 ** (0.13)**	**0.02 * (0.01)**	0.08 (0.07)	-0.02 (0.02)
Stream	**11.37 *** (3.22)**	-0.86 (0.52)	**-3.60 *** (0.72)**	0.31 (0.23)	-0.02 (0.01)	0.05 (0.12)	-0.04 (0.04)
R^2	0.52	0.19	0.63	0.23	0.18	0.06	0.15

Table S4: Linear models predicting the relative abundance of classes within the non-universal compound pool. Values in cells are mean estimated effects ± standard error relative to the intercept, with the intercept expressed relative to zero. Bolded values were statistically significant at ***p < 0.001, **p < 0.01, *p < 0.05. For all, degrees of freedom = 57.

Coefficient	Lignins	Tannins	Condensed Hydrocarbons	Proteins	Amino Sugars	Carbohydrates	Lipids	Unsaturated Hydrocarbons
Intercept: Site C1, 5 cm depth, shoulder position	**19.35 *** (1.38)**	**4.71 *** (0.58)**	**12.26 *** (2.52)**	**1.07 ** (0.37)**	**0.33 *** (0.08)**	**1.69 *** (0.30)**	**0.74 ** (0.23)**	**0.56 *** (0.07)**
Site C2	-0.86 (0.87)	-0.55 (0.36)	-1.80 (1.59)	0.19 (0.23)	-0.03 (0.05)	0.15 (0.19)	0.06 (0.14)	0.04 (0.05)
Site H1	**1.91 * (0.87)**	-0.35 (0.36)	-2.72 (1.59)	0.42 (0.23)	0.05 (0.05)	0.01 (0.19)	0.20 (0.14)	-0.01 (0.05)
Site H2	**3.00 ** (0.87)**	-0.26 (0.36)	**-3.55 * (1.59)**	0.44 (0.23)	**0.10 * (0.05)**	0.37 (0.19)	0.05 (0.14)	-0.04 (0.05)
15 cm	1.58 (0.90)	-0.45 (0.38)	-2.13 (1.64)	-0.26 (0.24)	**-0.18 *** (0.05)**	0.24 (0.20)	0.11 (0.15)	**0.14 ** (0.05)**
30 cm	**2.52 ** (0.90)**	**-2.08 *** (0.38)**	**-9.39 *** (1.64)**	0.39 (0.24)	**-0.15 ** (0.05)**	**0.80 *** (0.20)**	**0.60 *** (0.15)**	**0.27 *** (0.05)**
60 cm	**3.27 *** (0.90)**	**-2.03 *** (0.38)**	**-10.89 *** (1.64)**	-0.28 (0.24)	**-0.20 *** (0.05)**	**0.67 ** (0.20)**	0.16 (0.15)	**0.15 ** (0.05)**
Backslope	0.95 (0.90)	-0.26 (0.38)	-1.84 (1.64)	0.44 (0.24)	0.07 (0.05)	-0.13 (0.20)	0.29 (0.15)	-0.09 (0.05)
Footslope	**3.67 *** (0.90)**	**-1.01 ** (0.38)**	**-5.14 ** (1.64)**	**0.68 ** (0.24)**	0.05 (0.05)	0.27 (0.20)	**0.34 * (0.15)**	**-0.10 * (0.05)**
Toeslope	**2.47 ** (0.90)**	**-1.75 *** (0.38)**	**-7.33 *** (1.64)**	**0.97 *** (0.24)**	0.10 (0.05)	0.19 (0.20)	**0.61 *** (0.15)**	-0.09 (0.05)
Stream	**5.49 *** (1.52)**	**-1.92 ** (0.64)**	**-11.36 *** (2.78)**	0.01 (0.41)	**-0.22 * (0.08)**	0.63 (0.33)	0.35 (0.25)	0.02 (0.08)
R^2	0.43	0.50	0.56	0.27	0.29	0.24	0.30	0.32

Table S5: Linear models predicting the intensity-weighted molecular mass of classes within the non-universal compound pool. Values in cells are mean estimated effects ± standard error relative to the intercept, with the intercept expressed relative to zero. Bolded values were statistically significant at ***p < 0.001, **p < 0.01, *p < 0.05. For all, degrees of freedom = 57.

Coefficient	Lignins	Tannins	Condensed Hydrocarbons	Proteins	Amino Sugars	Carbohydrates	Lipids	Unsaturated Hydrocarbons
Intercept: Site C1, 5 cm depth, shoulder position	**519.14 *** (12.17)**	**471.46 *** (15.51)**	**447.88 *** (18.92)**	**473.10 *** (16.79)**	**417.61 *** (19.66)**	**430.64 *** (18.47)**	**342.81 *** (12.66)**	**282.17 *** (10.87)**
Site C2	-6.26 (7.70)	-5.01 (9.81)	-8.30 (11.97)	-12.18 (10.62)	-7.81 (12.44)	-0.34 (11.68)	-4.23 (8.01)	-1.89 (6.88)
Site H1	**-18.34 * (7.70)**	7.16 (9.81)	-10.67 (11.97)	**-30.42 ** (10.62)**	-9.44 (12.44)	16.19 (11.68)	**-27.28 ** (8.01)**	4.53 (6.88)
Site H2	**-18.49 * (7.70)**	3.83 (9.81)	-2.27 (11.97)	-20.47 (10.62)	3.60 (12.44)	**28.44 * (11.68)**	**-18.83 * (8.01)**	10.69 (6.88)
15 cm	-2.29 (7.94)	7.15 (10.11)	-9.57 (12.34)	19.18 (10.94)	9.34 (12.82)	21.62 (12.04)	**27.93 ** (8.25)**	9.84 (7.09)
30 cm	**-17.56 * (7.94)**	-10.52 (10.11)	**-36.71 ** (12.34)**	-21.29 (10.94)	**-28.73 * (12.82)**	**54.31 *** (12.04)**	**34.96 *** (8.25)**	13.64 (7.09)
60 cm	-8.40 (7.94)	7.94 (10.11)	1.11 (12.34)	6.78 (10.94)	0.01 (12.82)	**72.43 *** (12.04)**	**31.79 *** (8.25)**	**36.91 *** (7.09)**
Backslope	-1.24 (7.94)	5.09 (10.11)	11.30 (12.34)	-4.02 (10.94)	3.46 (12.82)	20.29 (12.04)	15.70 (8.25)	**12.45 (7.09)**
Footslope	-8.12 (7.94)	12.82 (10.11)	3.65 (12.34)	**-24.83 * (10.94)**	1.77 (12.82)	**53.12 *** (12.04)**	4.87 (8.25)	**26.47 *** (7.09)**
Toeslope	**-25.69 ** (7.94)**	-0.23 (10.11)	-8.91 (12.34)	**-61.28 *** (10.94)**	-18.63 (12.82)	**65.37 *** (12.04)**	4.96 (8.25)	**21.86 ** (7.09)**
Stream	-11.00 (13.46)	18.84 (17.14)	12.18 (20.92)	9.43 (18.55)	16.31 (21.73)	**102.10 *** (20.42)**	**47.08 ** (13.99)**	**48.16 *** (12.02)**
R^2	0.21	-0.02	0.10	0.46	0.10	0.55	0.33	0.39

Table S6: Linear models predicting the molecular mass of dissolved organic matter. Molecular mass (Da) is equivalent to the mass to charge ratio (m/z) because all molecules are singly charged. Values in cells are mean estimated effects ± standard error relative to the intercept, with the intercept expressed relative to zero. Bolded values were statistically significant at ***p < 0.001, **p < 0.01, *p < 0.05. For all, degrees of freedom = 57.

Coefficient	**Intensity-Weighted Mass (All Compounds)**	**Mass (All Compounds)**	**Mass (Non-Universal Compounds)**
Intercept: Site C1, 5 cm depth, shoulder position	**437.94 *** (8.15)**	**476.77 *** (8.33)**	**491.24 *** (9.62)**
Site C2	-4.27 (5.16)	-7.07 (5.27)	-7.02 (6.08)
Site H1	-0.37 (5.16)	-7.19 (5.27)	-7.88 (6.08)
Site H2	1.47 (5.16)	-4.29 (5.27)	-4.32 (6.08)
15 cm	4.04 (5.31)	-1.03 (5.43)	-1.62 (6.27)
30 cm	-1.60 (5.31)	**-11.63 * (5.43)**	**-13.68 * (6.27)**
60 cm	10.70 * (5.31)	-3.53 (5.43)	-2.13 (6.27)
Backslope	4.77 (5.31)	2.90 (5.43)	3.93 (6.27)
Footslope	10.24 (5.31)	3.00 (5.43)	3.54 (6.27)
Toeslope	-0.29 (5.31)	-8.45 (5.43)	-9.85 (6.27)
Stream	**18.43 * (9.01)**	2.97 (9.21)	4.90 (10.63)
R^2	0.07	0.07	0.08

Table S7: Linear models predicting changes in environmental variables at each depth and hillslope position at each site. Values in cells are mean estimated effects on a log-scale ± standard error relative to the intercept, with the intercept expressed relative to zero. Bolded values were statistically significant at ***p < 0.001, **p < 0.01, *p < 0.05. For all, degrees of freedom = 57.

Coefficient	DOC (mg L^{-1})	Bacterial Productivity (10^{-3} mg C L^{-1})	Humic Substances: Low Molecular Weight Substances	Carbon: Nitrogen (Total)
Intercept: Site C1, 5 cm depth, shoulder position	**4.98 *** (1.23)**	**0.01 *** (0.01)**	**2.33 * (0.80)**	**22.20 *** (7.16)**
Site C2	1.00 (0.13)	NA	1.12 (0.21)	0.72 (0.12)
Site H1	**0.51 *** (0.07)**	**2.40 * (0.95)**	**0.60 ** (0.11)**	**0.33 *** (0.06)**
Site H2	**0.52 *** (0.07)**	NA	0.74 (0.14)	**0.23 *** (0.04)**
15 cm	0.79 (0.11)	0.55 (0.30)	**1.50 * (0.28)**	**0.66 * (0.11)**
30 cm	**0.48 *** (0.06)**	0.38 (0.20)	0.91 (0.17)	**0.56 ** (0.10)**
60 cm	**0.28 *** (0.04)**	**0.12 ** (0.07)**	0.97 (0.19)	**0.46 *** (0.08)**
Backslope	**0.74 * (0.10)**	2.70 (1.41)	0.86 (0.16)	0.83 (0.14)
Footslope	**0.33 *** (0.04)**	**4.83 ** (2.63)**	**0.51 *** (0.09)**	**0.66 * (0.12)**
Toeslope	**0.20 *** (0.03)**	**0.02 *** (0.01)**	**0.39 *** (0.08)**	0.80 (0.14)
Stream	**8.40 *** (2.15)**	NA	**2.20 * (0.78)**	1.75 (0.58)
R^2	0.84	0.78	0.42	0.63

Table S8: List of measured environmental variables. Variables were grouped by four categories (top row) corresponding to Fig. 4. Numbers in brackets following the CAZyme family name refer to, first, the number of individual CAZYme subfamilies included in our analyses and, second, the number that were important for explaining dissolved organic matter (DOM) composition across the 8 compound classes based on the redundancy analysis (RDA) inferential framework (see Methods). Bolded variables indicate the 62 environmental variables that were important for explaining DOM composition across the 8 compound classes. Bacterial and fungal taxonomic diversities were assessed using exact sequence variants (ESVs) generated by amplicon sequencing of the 16S rRNA gene and ITS2 region, respectively.

Expression of CAZymes (n=412, bold=51)	Enzyme Activity and Biomass (n=38, bold=6)	Microbial Diversity (n=6, bold=0)	Physical Chemistry (n=19, bold=4)
**auxiliary activity family (14,1)**	**2-hydroxy benzoic acid**	shannon diversity index (ITS)	**soil moisture content**
**carbohydrate esterase (16,6)**	4-hydroxy benzoic acid	Number of ESVs (16S)	**pH**
**carboydrate-binding module (37,1)**	**D, L-α-glycerol phosphate**	Number of ESVs (ITS)	conductivity
cohesin (1,0)	D-cellobiose	Shannon diversity index (16S)	alkalinity
dockerin (1,0)	D-dalactonic acid γ-lactone	Jaccard index (16S)	calcium
**glycoside hydrolase (229,27)**	D-galacturonic acid	Jaccard index (ITS)	potassium
**glycosyl transferase (70,9)**	D-glucosaminic acid		magnesium
**polysaccharide lyase family (43,7)**	D-malic acid		sodium
s-layer homology (1,0)	D-mannitol		sulphate
	D-xylose		chlorine
	glucose-1-phosphate		silicon dioxide
	glycogen		nitrate
	glycyl-L-glutamic acid		ammonium
	itaconic acid		total inorganic carbon
	L-Arginine		**aluminum**
	L-Asparagine		iron
	l-Erythritol		manganese
	L-Phenylalanine		zinc
	L-Serine		**total nitrogen**
	L-Threonine		
	N-acetyl-D-glucosamine		
	N-acetylglucosaminidase		
	phenylethylamine		
	**phosphatase**		
	putrescine		

pyruvic acid methyl ester		
**tween 40**		
tween 80		
xylanase		
α-cyclodextrin		
α-D-lactose		
α-ketobutyric acid		
**β-glucosidase**		
**β-methyl-D-glucoside**		
γ-hydroxybutyric acid		
microbial biomass		
basal respiration		

Table S9: Differentially expressed genes annotated as carbohydrate-active enzymes (CAZymes). We separately predicted the expression of 412 CAZymes from soil depth, hillslope position, and catchment identity and accounted for repeated measurement of the same soil core using mixed effects models with negative binomial error structures (see Main Text). We used these models to estimate the statistical significance of changes in marginal means from 5 to 60 cm averaged across catchments and hillslope positions, and from shoulder to toeslope positions averaged across catchments and depths, using the R package emmeans[8]. Here we report the list of CAZymes that varied from 5 to 60 cm and/or shoulder to toeslope and that also explained variation in non-universal compound classes that increased in molecular mass along across the depth and hillslope gradients, i.e., bolded classes in Fig 4b. Two-sided Wald $\chi 2$ tests report the overall statistical significance of predictors, and p values were adjusted for multiple comparisons with a false discovery rate (q-value) <0.05 considered statistically significant[9]. We sorted CAZymes from the smallest to largest mean q-value. *q <0.05, **q <0.01, ***q <0.001.

Carbohydrate-active enzyme (abbreviation)	Mean # copies	Depth $\chi 2$	Hillslope $\chi 2$	Catchment $\chi 2$
Glycoside Hydrolase Family 23 (GH23)	34708.6	16.2 **	10.1 **	8.0 **
Glycosyltransferase Family 2, Subfamily 3 (GT2_Glyco_tranf_2_3)	39386.3	6.2 **	33.1 ***	12.0 **
Glycoside Hydrolase Family 129 (GH129)	173.4	12.5 **	10.2 **	3.0 *
Glycoside Hydrolase Family 139 (GH139)	57.6	7.9 **	11.4 **	1.4
Polysaccharide Lyase Family 16 (PL16)	7.2	7.1 **	10.8 **	0.2
Glycosyltransferase Family 89 (GT89)	1500.3	13.1 **	7.9 *	6.2 *
Glycoside Hydrolase Family 16, Subfamily 24 (GH16_24)	10.3	7.6 **	9.1 *	4.5 *
Carbohydrate-Binding Module 2 (CBM2)	110.5	12.7 **	7.2 *	10.5 **
Auxiliary Activity Family 1 (AA1)	48946.4	7.7 **	6.8 *	11.8 **
Carbohydrate Esterase Family 15 (CE15)	1718.1	14.1 **	3.9 *	5.6 *
Glycoside Hydrolase Family 43, Subfamily 23 (GH43_23)	1.0	2.2 *	10.0 **	0.9
Glycoside Hydrolase Family 5, Subfamily 26 (GH5_26)	78.8	3.7 *	5.5 *	0.0
Polysaccharide Lyase Family 33, Subfamily 1 (PL33_1)	9.9	2.0 *	8.8 *	18.0 ***
Polysaccharide Lyase Family 1, Subfamily 2 (PL1_2)	1353.2	4.5 *	4.6 *	19.2 ***
Glycoside Hydrolase Family 43, Subfamily 17 (GH43_17)	3.4	9.6 **	2.8	1.1
Glycosyltransferase Family 12 (GT12)	10.1	1.8 *	3.3	2.8 *
Polysaccharide Lyase Family 21 (PL21)	11.3	6.6 **	1.1	6.9 **
Glycoside Hydrolase Family 51 (GH51)	7447.0	3.7 *	1.6	10.8 **
Glycoside Hydrolase Family 13, Subfamily 18 (GH13_18)	482.4	13.2 **	0.7	3.4 *
Glycoside Hydrolase Family 135 (GH135)	498.2	6.9 **	0.3	7.1 **

Table S10: Data sources used to generate Figure 5. The mean percentage of molecules shared with a deep-sea reference sample was calculated for different sample types. If there was more than one study for a given sample type, the number of samples from each study is given in parenthesis.

Sample Type	Number of Samples	Study
5 cm Depth (Soil)	19	this one, Simon et al.[11] (3)
15 cm Depth (Soil)	20	this one, Simon et al.[11] (4)
30 cm Depth (Soil)	17	this one, Simon et al.[11] (1)
60 cm Depth (Soil)	17	this one, Simon et al.[11] (1)
Shoulder (Soil)	16	this one
Backslope (Soil)	16	this one
Footslope (Soil)	16	this one
Toeslope (Soil)	16	this one
Stream	15	this one (4), Hutchins et al. 2017[12] (11)
River	144	Hutchins et al. 2017[12]
Lake	116	Kellerman et al. 2014[13](115) Zark & Dittmar 2018[14] (1)
Bog	4	Simon et al. 2018[11]
Sea Surface	4	Zark & Dittmar 2018[14]
Aquifer	2	Simon et al. 2018[11]
Deep-sea	4	Simon et al. 2018[11]

Table S11: Primer details for amplicon sequencing of the ITS2 and 16S rRNA regions. JGI is the joint genome institute (https://genome.jgi.doe.gov/portal/

**Target gene**	**Primer name**	**Sequence**	**Reference**
Fungal ITS2	ITS9F	GAACGCAGCRAAIIGYGA	Menkis et al., 2012[15,16] ; White et al., 1990[2]; used at JGI
	ITS4R	TCCTCCGCTTATTGATATGC	
Archaeal and Bacterial 16S rRNA V4-V5	515F-Y	GTGYCAGCMGCCGCGGTAA	Parada et al., 2015[17]; used at JGI
	926R	CCGYCAATTYMTTTRAGTTT	

Table S12: Summary of physical and chemical environmental variables measured in soil pore water. D.L. = Detection limit.

Parameter/Range/ Detection Limit	Procedure	Principal Equipment
pH (3 - 9)	Orion Ross Ultra glass Electrode	Man-Tech PC-Titrate, Orion Thermo combination pH electrode
Specific Conductance (0 - 150 umho)	PCE-96-CT1003 electrode us/cm @ 25 C	Man-Tech PC-Titrate, with 4510 Conductivity meter
Total Alkalinity (0 - 2 meq/l)	Electrometric Titration	Man-Tech PC-Titrate, Titra-Sip titrator, Orion Thermo combination pH electrode
Total Nitrogen (0 - 2 ppm) D.L. - 0.05ppm	Automated Cadmium Reduction	Technicon Autoanalyser II - NO2+ NO3 channel - Autoclave Digestion - N.A.P. software
NH4 as N (0 - 500ppb) D.L. - 10ppb	Automated Sodium Nitroprusside, Filtered .45um	Seal Analytical AA3 Autoanalyzer AACE 6.07 software
NO2+NO3 as N (0 - 2ppm) D.L. - .04ppm	Automated Cadmium Reduction, Filtered .45um	Seal Analytical AA3 Autoanalyzer AACE 6.07 software
Total Phosphorus (unfiltered) (0 - 60ppb) D.L. - 1ppb	Automated Molybdophosphoric Blue	Technicon Autoanalyser II - N.A.P. software - Autoclave Digestion
Potassium – low end accuracy (water) 0.01ppm	ICP-MS	Agilent 7700x Inductively Coupled Plasma Instrument - Masshunter software
Sodium - low end accuracy (water) 0.01ppm	ICP-MS	Agilent 7700x Inductively Coupled Plasma Instrument - Masshunter software
Calcium – low end accuracy (water) 0.01ppm	ICP-MS	Agilent 7700x Inductively Coupled Plasma Instrument - Masshunter software
Magnesium – low end accuracy (water) 0.01ppm	ICP-MS	Agilent 7700x Inductively Coupled Plasma Instrument - Masshunter software
Sulphate (0 - 10ppm) D.L. -.2ppm	Conductance - ion exchange suppression	Dionex ICS 1100 Ion Chromatograph- Chromeleon 7.0 software
Total Sulphur as Sulphate	ICP-MS	Agilent 7700x Inductively Coupled Plasma Instrument- Masshunter software

Chloride<br>(0 - 5ppm)<br>D.L. - .2 ppm	Conductance - Ion Exchange Suppression	Dionex ICS 1100 Ion Chromatograph-Chromeleon 7.0 software
Silica Oxide<br>(0 - 50ppm) & (0-7ppm)<br>D.L. - .25 ppm	Automated Ascorbic Acid	Technicon Autoanalyser II - N.A.P. software
Iron - low end accuracy (water) 0.005ppm	ICP-MS	Agilent 7700x Inductively Coupled Plasma Instrument - Masshunter software
Aluminum – low end accuracy (water) 0.005ppm	ICP-MS	Agilent 7700x Inductively Coupled Plasma Instrument - Masshunter software
Manganese - low end accuracy (water) 0.0005ppm	ICP-MS	Agilent 7700x Inductively Coupled Plasma Instrument - Masshunter software
Zinc – low-end accuracy (water) 0.001ppm	ICP-MS	Agilent 7700x Inductively Coupled Plasma Instrument - Masshunter software
Copper - low-end accuracy (water) 0.0005ppm	ICP-MS	Agilent 7700x Inductively Coupled Plasma Instrument - Masshunter software
Nickel – low-end accuracy (water) 0.0005ppm	ICP-MS	Agilent 7700x Inductively Coupled Plasma Instrument - Masshunter software
Cadmium – low-end accuracy (water) 0.0005ppm	ICP-MS	Agilent 7700x Inductively Coupled Plasma Instrument - Masshunter software
Lead – low-end accuracy (water) 0.0005ppm	ICP-MS	Agilent 7700x Inductively Coupled Plasma Instrument - Masshunter software
Dissolved Organic Carbon (0 - 20 ppm)<br>DL. - .4ppm	Sample Purged - N2 Acid & Potassium Persulphate - U.V. Rad. - Dialysis - O.C. Inversely Meas., Filtered .45um	Seal Analytical AA3 Autoanalyzer - AACE 6.07 software
Dissolved Inorganic Carbon (0 - 5ppm) DL. - .5ppm	Sample Acidified H2SO4 - CO2 Dialysis Thru Membrane - I.C. Inversely Meas., Filtered .45um	Seal Analytical AA3 Autoanalyzer - AACE 6.07 software
Soluble Reactive Phosphorus (0 - 60ppb)<br>D.L. - 1ppb	Automated Molybdophosphoric Blue, Filtered .45um	Technicon Autoanalyser II - N.A.P. software
High-level NH4 as N<br>(0 – 100ppm)<br>D.L. – 2ppm	Automated Sodium Nitroprusside	Technicon Autoanalyser II - N.A.P. software
Arsenic – low-end accuracy (water) 0.0005ppm	ICP-MS	Agilent 7700x Inductively Coupled Plasma Instrument - Masshunter software

Cobalt – low-end accuracy (water) 0.0005ppm	ICP-MS	Agilent 7700x Inductively Coupled Plasma Instrument - Masshunter software
Chromium – low end accuracy (water) 0.0005ppm	ICP-MS	Agilent 7700x Inductively Coupled Plasma Instrument - Masshunter software
Selenium – low end accuracy (water) 0.001ppm	ICP-MS	Agilent 7700x Inductively Coupled Plasma Instrument - Masshunter software
Strontium – low-end accuracy (water) 0.001ppm	ICP-MS	Agilent 7700x Inductively Coupled Plasma Instrument - Masshunter software

Supplementary Results

To assess processing of dissolved organic matter (DOM) further, we averaged molecular masses in each sample weighted by the relative intensity of formulas[18]. In soils, increasing mass is thought to reflect the transformation of small plant-derived compounds into larger degradation products by microbes[18]. As expected if DOM shifted from plant- to microbial-derived compounds[18], the weighted molecular mass increased by an estimated mean (95% CI) of 2.5% (<0.1 to 5.1%) from 422 (415 to 430) Da to 433 (426 to 441) Da from 5 to 60 cm (Table S3). This shift was much stronger from shoulder into stream positions, increasing by 10.1% (4.2 to 16.0%) from 422 (414 to 430) Da to 437 (422 to 452) Da (Table S6). Increases in molecular mass were less consistent when compounds were not weighted by their relative intensities (Table S6). Furthermore, averaged values of all compounds lose the strong underlying signal seen when examining individual compound classes (see Main Text), though this averaged signal is comparable to the 21 Da (6% change) reported in Roth et al.[9].

Supplementary Methods

Rarefaction of DOM: We combined published data from Table S9 into a sample × molecular formula matrix. We then randomly generated 10,000 intervals between 250 to 20078, and randomly selected the corresponding molecular formula from the sample set. We then plotted the proportion of universal compounds in each sample as a function of the total number of sampled formulae, repeating this process 999 times. We visually identified a threshold of 6000 molecular formulae, after which additional sampling did not change the proportion of universal compounds per sample.

Bacterial productivity: In the laboratory, 1 g of soil from each sample was mixed with 50 mL distilled water using a gyratory shaker (200 rev min^{-1}) and soil particles were removed by centrifugation (1000 *g*) for 10 min. We then added 3.74 µL of ^{3}H-labeled leucine (1 mCi mL^{-1}, PerkinElmer, USA) to 1.5 mL of bacterial suspension. Blanks were prepared by immediately adding 75 µL of 100% trichloroacetic acid (TCA), killing any live cells, and providing a measure of background ^{3}H leucine incorporation that was later subtracted from the incubated values. After 2 h of incubation at room temperature (~22°C), each sample received 75 µL of ice-cold 100% trichloroacetic acid (TCA) to stop incorporation. Samples were subsequently centrifuged at 13,000 *g* for 10 min and the supernatant was discarded. Each sample was then washed once with 5% TCA, 1.5 mL ice-cold 80% ethanol, and 0.2 mL 1M NaOH. Samples were vortexed and maintained at 90°C for 1 h. After cooling to room temperature, we added 1 mL of Ultima Gold scintillation fluid (Perkin Elmer, USA) to each

sample and vortexed for 30 s. ^3H incorporation was measured with a LS6500 liquid scintillation counter (Beckman Coulter, USA).

Bacterial cell counts: Each sample was defrosted and we added 1.2 mL of a detergent: 250 mM tetrasodium pyrophosphate [TSP; pH 8.0] containing Tween 80, 0.5% final concentration. The solution was then vortexed for 30 s and shaken for 2 hr at 4°C. From the resulting slurry, 1 mL was layered slowly onto 0.5 mL of Histodenz solution (80% [wt/vol] prepared in 50 mM sterile TSP buffer). Bacterial cells and soil particles were separated by high-speed centrifugation (14,000 *g*) for 30 min. The upper and middle cell-containing phases (including the thin layer on top of the Histodenz cushion liquid phase) were carefully recovered and mixed with 1 mL of 50 mM TSP buffer and centrifuged at 17,000 *g* for 25 min. The supernatant was removed, and the cell pellet containing the cell fraction was resuspended in 0.8 ml of 50mM TSP buffer. The pellet slurries were vortexed and then stained with SYBR Green (final concentration 1×) and incubated for 20 min at room temperature in the dark. Samples were vortexed again before cytometry counts, and between each sample, 100 µL of sterile TSP buffer was passed through the flow cytometer.

Principal coordinates of neighbour matrices: The distance between the lysimeters was represented as a Euclidean distance matrix. Distances were calculated from the average latitude and longitude of the three replicate lysimeters at the same hillslope position. The resulting PCNM decomposes any spatial relationships between sample locations at the same site. This captures distinct information which justifies its inclusion alongside the ordinal variables (depth) and categorical variables (hillslope). We did not compute distances between lysimeters in different catchments by setting a threshold distance of 500 m[19].

Supplementary References

1. Kellerman, A. M. *et al.* Unifying Concepts Linking Dissolved Organic Matter Composition to Persistence in Aquatic Ecosystems. *Environ. Sci. Technol.* **52**, 2538–2548 (2018).
2. Behnke, M. I. *et al.* Pan-arctic riverine dissolved organic matter: Synchronous molecular stability, shifting sources and subsidies. *Global Biogeochem. Cycles* **35**, (2021).
3. Hertkorn, N. *et al.* Characterization of a major refractory component of marine dissolved organic matter. *Geochim. Cosmochim. Acta* **70**, 2990–3010 (2006).
4. Kim, S., Kramer, R. W. & Hatcher, P. G. Graphical method for analysis of ultrahigh-resolution broadband mass spectra of natural organic matter, the van Krevelen diagram. *Anal. Chem.* **75**, 5336–5344 (2003).
5. Merder, J. *et al.* ICBM-OCEAN: Processing Ultrahigh-Resolution Mass Spectrometry Data of Complex Molecular Mixtures. *Anal. Chem.* **92**, 6832–6838 (2020).
6. Lechtenfeld, O. J. *et al.* Molecular transformation and degradation of refractory dissolved organic matter in the Atlantic and Southern Ocean. *Geochim. Cosmochim. Acta* **126**, 321–337 (2014).
7. Bartoń, K. MuMIn: Multi-modal inference. Model selection and model averaging based on information criteria (AICc and alike). http://cran.r-project.org/web/packages/MuMIn/index.html (2013).
8. Lenth, R. emmeans: estimated marginal means, aka least-squares means. R package version 1.4. 7. 2020.

9. Rivellese, F. *et al.* Rituximab versus tocilizumab in rheumatoid arthritis: synovial biopsy-based biomarker analysis of the phase 4 R4RA randomized trial. *Nat. Med.* **28**, 1256–1268 (2022).
10. Love, M. I., Huber, W. & Anders, S. Moderated estimation of fold change and dispersion for RNA-seq data with DESeq2. *Genome Biol.* **15**, 550 (2014).
11. Simon, C., Roth, V.-N., Dittmar, T. & Gleixner, G. Molecular Signals of Heterogeneous Terrestrial Environments Identified in Dissolved Organic Matter: A Comparative Analysis of Orbitrap and Ion Cyclotron Resonance Mass Spectrometers. *Front Earth Sci. Chin.* **6**, (2018).
12. Hutchins, R. H. S. *et al.* The optical, chemical, and molecular dissolved organic matter succession along a boreal soil-stream-river continuum. *J. Geophys. Res. Biogeosci.* **122**, 2892–2908 (2017).
13. Kellerman, A. M., Dittmar, T., Kothawala, D. N. & Tranvik, L. J. Chemodiversity of dissolved organic matter in lakes driven by climate and hydrology. *Nat. Commun.* **5**, 3804 (2014).
14. Zark, M. & Dittmar, T. Universal molecular structures in natural dissolved organic matter. *Nat. Commun.* **9**, 3178 (2018).
15. Menkis, A. *et al.* Occurrence and impact of the root-rot biocontrol agent Phlebiopsis gigantea on soil fungal communities in Picea abies forests of northern Europe. *FEMS Microbiol. Ecol.* **81**, 438–445 (2012).
16. White, T. J., Bruns, T., Lee, S. & Taylor, J. Amplification and direct sequencing of fungal ribosomal RNA genes for phylogenetics. in *PCR Protocols* 315–322 (Elsevier, 1990).
17. Parada, A. E., Needham, D. M. & Fuhrman, J. A. Every base matters: assessing small subunit rRNA primers for marine microbiomes with mock communities, time series and global field samples. *Environ. Microbiol.* **18**, 1403–1414 (2016).
18. Roth, V.-N. *et al.* Persistence of dissolved organic matter explained by molecular changes during its passage through soil. *Nat. Geosci.* **12**, 755–761 (2019).
19. Borcard, D. & Legendre, P. All-scale spatial analysis of ecological data by means of principal coordinates of neighbour matrices. *Ecological Modelling* vol. 153 51–68 (2002).
